# A Study on the Effect of Pd Layer Thickness on the Properties of Cu-Ag Intermetallic Compounds at the Bonding Interface

**DOI:** 10.3390/ma17174335

**Published:** 2024-09-01

**Authors:** Junling Fan, Donglin Yuan, Juan Du, Tao Hou, Furong Wang, Jun Cao, Xuemei Yang, Yuemin Zhang

**Affiliations:** 1 School of Chemical and Environmental Engineering, Jiaozuo University, Jiaozuo 454000, China; hgxybgs@126.com (D.Y.); juandj@126.com (J.D.); houtao@jzu.edu.cn (T.H.); yxm01@126.com (X.Y.); 2 School of Mechanical and Power Engineering, Henan Polytechnic University, Jiaozuo 454000, China; wangfr@hpu.edu.cn (F.W.); cavan@hpu.edu.cn (J.C.); zhangyuemin@hpu.edu.cn (Y.Z.)

**Keywords:** Pd-coated Cu wires, Cu-Ag intermetallic compounds

## Abstract

This paper conducted a high-temperature storage test (HTST) on bonded samples made of Pd100 (Pd-coated Cu wire with a Pd layer thickness of 100 nm) and Pd120, and studied the growth law of Cu-Ag intermetallic compounds and the inhibitory mechanism of Pd thickness on Cu-Ag intermetallic compounds. The results show that the Kirkendall effect at the bonding interface of the Pd100-bonded sample is more obvious after the HTST, the sizes of voids and cracks are larger, and the thickness of intermetallic compounds is uneven. But, the bonding interface of the Pd120-bonded sample has almost no microcracks, the Kirkendall voids are small, and the intermetallic compound size is uniform and relatively thin. The formation sequence of intermetallic compounds is as follows: Cu atoms diffuse into the Ag layer to form Ag-rich compounds such as CuAg_4_ or CuAg_2_, and then the CuAg forms with the increase in diffused Cu elements. Pd can significantly reduce the Kirkendall effect and slow down the growth of Cu-Ag intermetallic compounds. The growth rate of intermetallic compounds is too fast when the Cu bonding wire has a thin Pd layer, which results in holes and microcracks in the bonding interface and lead to the peeling of the bonding interface. Voids and cracks will hinder the continuous diffusion of Cu and Ag atoms, resulting in the growth of intermetallic compounds being inhibited.

## 1. Introduction

Bond wire is an important structural material in microelectronic packaging, playing a crucial role in connecting integrated circuit chips and external lead frames [1]. Copper and its alloys are widely used in fields such as the electronics industry, high-energy facilities, railway transportation, etc., due to their excellent electrical conductivity, thermal conductivity, corrosion resistance, ease of processing, and cost effectiveness [2,3,4]. Cu has been one of the most used bonding wire materials in the present day due to its outstanding performance and cost competitiveness [5,6,7]. Several studies have shown the bonding reliability of copper wire under various conditions, including high-temperature storage, thermal cycling, humidity, and thermal shock [8,9,10,11,12,13]. The use of Cu bonding wires is, however, plagued by the major drawback of poor reliability that results from the corrosion of the intermetallic compounds (IMCs) at the bonding interface [14,15,16,17]. Therefore, materials such as Au, Ag, Al, Ni, and Pd have been proposed for coating on Cu wires to improve the corrosion resistance and reliability [18,19,20,21]. There is evidence that Pd-coated Cu (PCC) bonding wires have become a second alternative to Au wires, to address the reliability issue [22]. Furthermore, the Pd coating can suppress the occurrence of corrosion during the interfacial reaction between the ball bond and Al pad. In recent years, PCC has rapidly entered the market and been widely used in micro-pitch bonding. It has a longer shelf life than bare Cu wire due to the noble metal coating and is believed to have enhanced bond reliability under humid and electrically biased conditions [15]. IMCs at the bonding interface are the key factors in determining bonding quality. Many scholars have studied IMCs at the bonding interface of PCC, such as Adeline B.Y. Lim et al., who investigated the Pd distribution and grain structure of the free air ball (FAB) of PCC. The results indicate that the distribution of Pd in FAB is significantly influenced by the Electronic Flame Offs (EFOs) current and the type of covering gas used, with the EFOs current being the main factor [23]. Cheng et al. demonstrated through experiments that the shape of the FAB formed by PCC leads to a higher yield of 96% at moderate current settings [24]. Wentao Qin conducted a uHAST using ø20 μm Cu wire and PCC. The results showed that the Pd coating promoted the formation of Cu-rich IMCs in (CuPdx)Al and inhibited the formation of Cu-rich IMCs in (CuPdx)3Al2. In addition, IMCs with high (Cu + Pd)/Al atomic ratios corroded faster than IMCs with low atomic ratios [15,16,17,25].

Early studies have shown that the IMCs of bare Cu wire are hard and brittle [26,27,28], but in the IMCs of PCC wire, due to the small difference in electrical properties between Pd and Cu [29,30], a solid solution with a face-centered cubic structure is more easily formed, so the IMCs of PCC wire exhibit better ductility and a slower growth rate [31]. Both the quality and reliability of bonding are directly and closely related to IMCs, because the essence of dissimilar metal bonding is the formation of IMCs through atomic diffusion at the bonding interface and connect them together. Many studies have focused on the interfacial reactions between the Cu ball bond and the Al pad, as well as the growth behavior of Cu-Al IMCs [32,33,34]. However, there is little research on the IMCs between Cu and Ag. This article uses Cu wires with two different Pd thickness coatings for bonding and investigates the effects of Pd on the types and growth of IMCs between Cu and Ag after HTSTs. This provides a theoretical basis for the choice of Pd layer thickness for PCC wire and for predicting the reliability and working life of bonding points.

## 2. Experimental Materials and Methods

**Figure 1:**
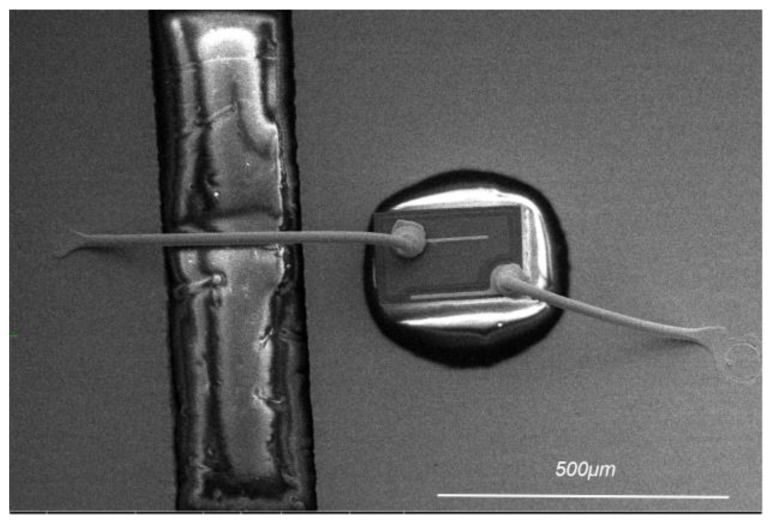
Morphology of bonded samples.

The experimental materials were PCC with two different Pd layer thicknesses (100 nm and 120 nm) (for ease of reference, they are named Pd100 and Pd120, respectively), and the Cu core diameter was 25 μm. Two types of PCC were subjected to bonding tests on the KAIJO FB-988 bonding machine (Made by KAIJO Corporation in Osaka, Japan). Orthogonal experiments were used to determine the optimal parameter range of bonding pressure and ultrasonic power. And, bonded wire samples were produced under their respective optimal bonding parameters, as shown in Figure 1.

**Figure 2:**
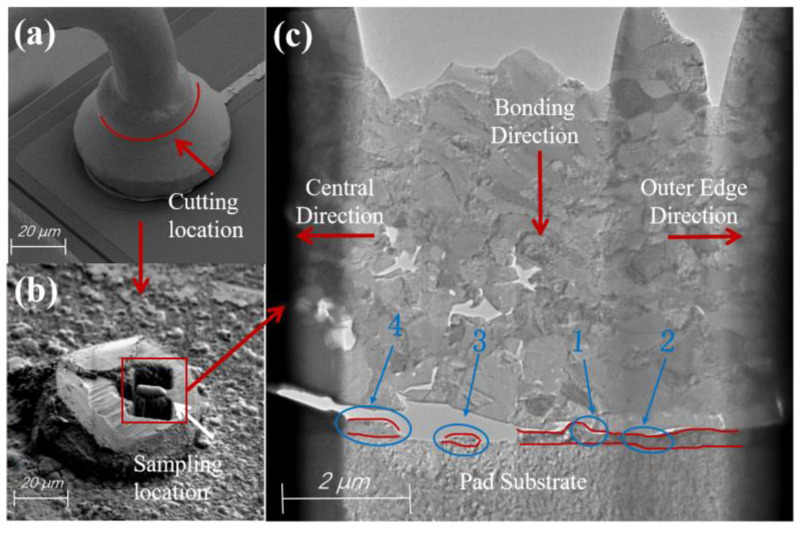
Sampling position and sample morphology of Pd100-bonded ball. (**a**) Original morphology of ball bonding points; (**b**) TEM sampling location; (**c**) TEM sample morphology. 1, 2, 3, and 4 are the areas that need to be tested.

Reliability tests were conducted on the bonded samples in a THP100 programmable constant temperature and humidity chamber. The samples were kept at 150 °C for 96 h and then removed. All regions above the shoulder of the bonded ball (Figure 2a) were cut using a focused ion beam (FIB), leaving only the ball-bonded point as shown in Figure 2b. Then, a thin slice was cut longitudinally from the ball bonded point shown in Figure 2b to create a sample, and the final result is shown in Figure 2c. Four regions in Figure 2c were selected for TEM and EDS experiments, and the types and proportions of elements at different positions within each region were counted. The formation mechanism of Cu-Ag intermetallic compounds (IMCs) was analyzed, and their growth patterns under high-temperature conditions have been studied.

## 3. Results and Discussion

### 3.1. Study of Pd100 IMCs

From Figure 2c, it can be seen that there are obvious voids inside the Cu bonded ball matrix, and there are wide cracks at the bonding interface. The pores and cracks near the center area are larger in size. After a long-term high-temperature storage test, a large number of IMCs grew at the bonding interface. Due to the Kirkendall effect, the Cu atoms quickly migrated to the Ag region [35]. The fast diffusion rate of Cu atoms led to the formation of voids [36,37]. Kirkendall voids caused by an imbalanced diffusion flux always formed on the side of the metal with higher diffusion rates, and an increase in the number of voids led to the generation of microcracks [38].

**Figure 3:**
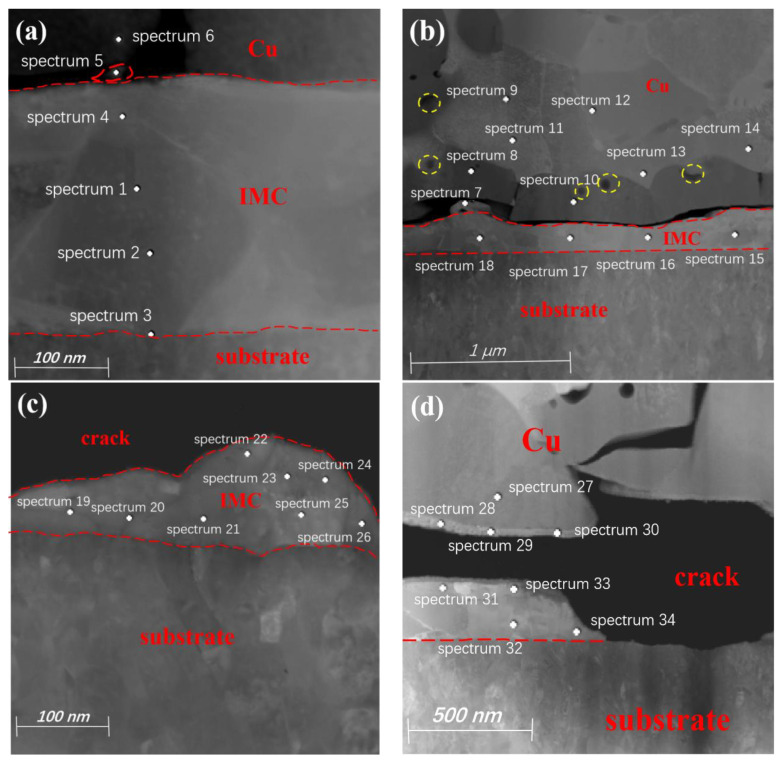
IMC morphology and detection positions in different regions of the Pd100-bonded sample. (**a**) Defect-free IMC; (**b**) the bonding interface has microcracks and pores appear in the Cu matrix; (**c**) inconsistent thickness of IMC; (**d**) large cracks appear in the bonds, interfaces, and Cu matrix.

The IMC morphology of region 1 in Figure 2c is shown in Figure 3a, where the IMCs have uniform thickness and no obvious cracks. From the EDS point-scan positions 1–4 in Table 1, it can be seen that the main component of the IMC is CuAg_2_, while the main component at position 5 is CuAg. Position 3 is closest to the pad of the substrate layer. According to the detection data, the entire Ag layer participated in the formation of IMCs after a long period of HTST. The formation mechanism of IMCs is as follows: during the bonding process, the bonded ball undergoes deformation with the help of pressure and ultrasonic energy. The atoms at the bottom of the bonded ball and at the surface of the pad penetrate and fuse with each other to form metallic compounds and a high-strength connection. 

Because only a small amount of Cu diffuses and participates in the reaction initially, the IMC formed is CuAg_2_. The diffusion rate of Cu atoms at the bonding interface is relatively fast, and they can quickly diffuse along dislocation channels to the Ag layer during the HTST process. As more Cu atoms keep diffusing to bond with the Ag, the Ag layer becomes fully involved in the reaction. The excess Cu atoms in turn combine with the CuAg_2_ to form CuAg, so the IMC at the position farther from the bonding interface is CuAg_2_. The IMC at position 5 is CuAg because of the abundance of Cu elements at the position closer to the bonding interface.

There are long microcracks between the IMCs of region 2 of the Cu bonded ball, and many voids appear close to the bonding interface, as shown in Figure 3b. From the analysis of the elemental content at positions 7–14, it can be seen that Pd exists inside the Cu ball, but it is not uniformly distributed, and the Pd content is lower on the bonded ball side near the Ag layer position. Some of the Pd atoms can be incorporated into the FAB during EFO, but no Pd is present at the bottom of the FAB. The formation of Pd-rich zones is due to the inability of both Pd and Cu to form a homogeneous solid solution at various EFO current settings. The Pd-rich phase is mainly concentrated in the neck region of the FAB, where some Pd is injected into the FAB body, while there is less Pd distribution near the tip [23]. The melting points for Cu and Pd are 1083 °C and 1554 °C, respectively, which means that Cu will melt first under high current setting, and Pd will melt later due to its higher melting point, resulting in an uneven distribution of the latter in FAB [39].

From the elemental content at positions 15–18, it can be seen that the main component of the IMCs is CuAg_2_ because Cu will first diffuse into the Ag layer to form CuAg_2_ in HTST, followed by the generation of Kirkendall voids. Due to the higher diffusion rate of Cu compared to Ag, and the thinner, unevenly distributed Pd coating of Pd100, the hindering effect on Cu diffusion is lighter. Therefore, the Kirkendall effect on the bonded ball side is more pronounced, and the hole density is higher than that of the Ag pads. The hole density at the bonding interface is larger and connected together to form microcracks, which block the continuous diffusion of Cu and Ag atoms, so the final IMC is primarily composed of CuAg_2_.

The IMC in region 3 is ‘island-shaped’ as shown in Figure 3c, and has a wide crack between it and the Cu bonded ball. According to the analysis of data from positions 19–26 in Table 1, the main component of the IMCs is CuAg. At the beginning of HTST, Cu atoms diffuse rapidly into the Ag layer by virtue of dislocation motion to form CuAg_2_. Since there is no Pd at the bonding interface in region 3, the center region of the bonding interface is not blocked by Pd, and the Cu atoms diffuse faster and combine with CuAg_2_ to form CuAg. The Kirkendall voids generated by Cu diffusion increase and connects together to form microcracks, which leads to the inability of Cu atoms to continue to diffuse, and thus the composition of the region 3 IMC is mainly CuAg.

**Figure 4:**
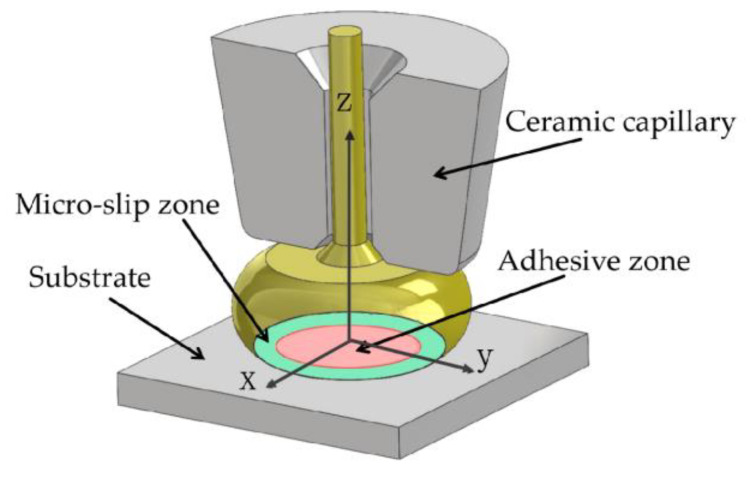
Partition of bonding interface [40].

The IMC in region 4 is discontinuous and there is a large crack between it and the Cu bonded ball, as shown in Figure 3d. From the atomic contents of positions 31–34, it can be seen that the main component of the IMC in region 4 is also CuAg, and the formation and transformation processes are almost the same as those in region 3. The Pd has a low concentration on the side of the Cu bonded ball near the bonding interface, and it exists almost exclusively in the interior of the Cu bonded ball. The closer to the center of the bonding region, the lower the Pd content, which also weakens the inhibitory effect of Pd on the growth of the IMC. In addition, region 4 is closest to the center of the bonding region, which is in an adhesive state, and the connection is not tight enough compared to the micro-slip zone [40] as shown in Figure 4. These factors, combined with the Kirkendall voids caused by the diffusion of Cu atoms, ultimately lead to the formation of microcracks.

In summary, the quality of the bonding interface in the Pd100-bonded sample deteriorates, and microcracks are generated when it is stored at a high temperature of 150 °C for 96 h, and the main components of the IMCs are CuAg_2_ and CuAg. Based on the distribution and elemental content of these two compounds, it can be inferred that Cu atoms first diffuse to the Ag pad at high temperatures to form CuAg_2_, and then continue to combine with Cu to form CuAg. When a Cu atom diffuses to a certain extent at the bonding interface, voids and cracks will be generated, which will hinder the growth and evolution of IMCs. There is no Pd element in the IMCs, and the Pd content decreases the closer it is to the Ag pad. The position at the center of the bonding region has the lowest Pd content. On the contrary, the position near the periphery of the bonding region has a higher Pd content. This is because the Pd atoms are mostly enriched in the upper spherical surface of the FAB during the burn-in stage, and less so in the interior and bottom. On the other hand, the Pd atoms will move from the interior of the matrix to the peripheral region with the extension of the HTST time, causing a decrease in Pd in the central region.

### 3.2. Study of Pd120 IMCs

**Figure 5:**
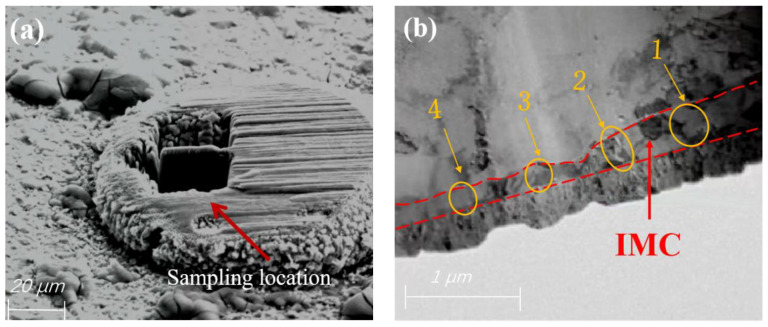
The sampling location and microscopic morphology of the Pd120-bonded ball. (**a**) the TEM sampling location; (**b**) the TEM sample morphology. 1, 2, 3, and 4 are the areas that need to be tested.

In Figure 5a, a thin slice was cut out to be used as TEM and EDS test samples, and the sampling results are shown in Figure 5b, and four regions were selected to analyze the IMC composition. From Figure 5b, it can be seen that there are no obvious holes inside the Cu bonded ball matrix and no wider cracks at the bonding interface.

**Figure 6:**
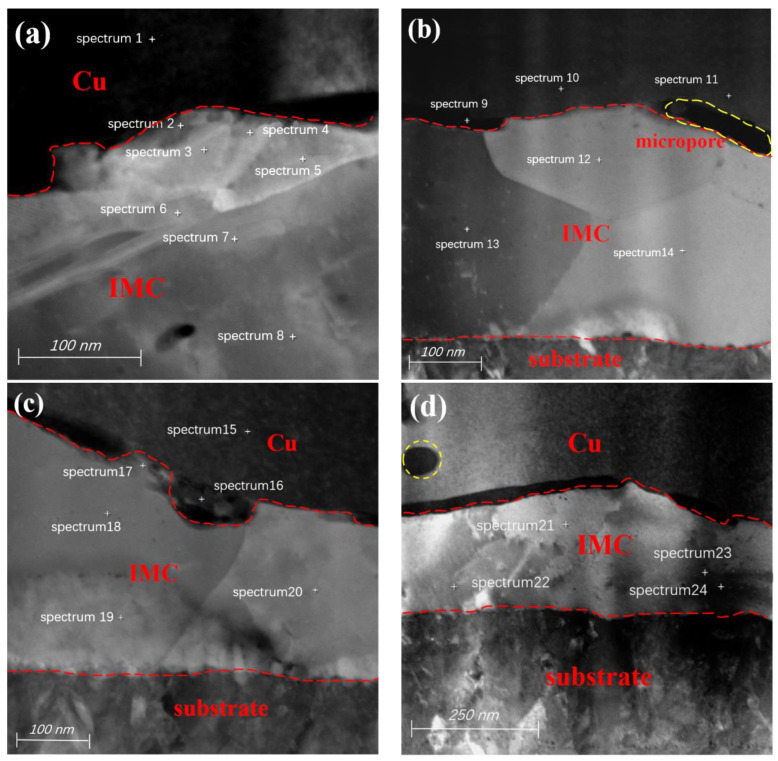
IMC morphology and detection position in different regions of the Pd120-bonded sample. (**a**–**d**) sites that correspond to detection positions 1, 2, 3, and 4, respectively.

The analyzed positions of region 1 are shown in Figure 6a. Position 1 in Table 2 shows that some of the Pd elements are solidly dissolved into the Cu matrix during the ball burn-in stage. The atomic content at position 2 shows that the main component of the IMC is CuAg, while the main component of the IMC at positions 3–8 is CuAg_4_.

Because Pd120 has a thicker Pd coating, it has a stronger hindering effect on the diffusion of Cu atoms, resulting in a reduction in Cu atoms diffusing into the Ag layer. So in the initial stage, the copper and Ag bond interface forms CuAg. In addition, the microcracks and holes formed by the Kirkendall effect at the bonding interface also hinder the further diffusion of Cu atoms, so the IMC composition on the side away from the bonded ball is CuAg_4_. The Ag coating near the side of the bonded ball can come into contact with abundant Cu elements, and the hindering effect of Pd is relatively small, so Cu and Ag atoms can fully come into contact, and its IMC component is CuAg. The microcracks at the bonding interface of the Pd120-bonded sample are narrower than those in Pd100, so the Pd elements do play a role in slowing down the rate of IMC and microcrack generation.

In Figure 6b, there are micropores between the IMC in region 2 and the bonded ball, and the presence of Pd is not detected at positions 9–11. The atomic content of positions 12–14 proves that the main component of the IMC in this region is CuAg_3_. Because the bonding interface has less Pd elements, the Cu atoms initially diffuse into the Ag layer to form CuAg_4_, and then slowly combine with Cu to form CuAg_3_ in a high-temperature environment. In the same environment and time, different IMCs are formed in region 1 and 2, which indicates that the IMC generation rate in different regions of the bonding interface is different.

The element Pd was detected at positions 15 and 16 in region 3 of Figure 6c. Because Pd has an inhibitory effect on the growth of IMCs, the reduction in Cu atoms results in the IMC composition at positions 18–20 being mainly composed of an Ag-rich CuAg_4_ phase. The main component of the IMC at position 17 is Cu_4_Ag, as this position allows for sufficient contact with Cu elements and allows some Ag atoms to reverse penetrate into the bonding interface.

From the atomic content of region 6 in Figure 6d, and EDS point-scanning positions 21 and 22 in Table 2, it can be seen that the IMC composition in this region is mainly CuAg_2_, while the IMC composition of points 23 and 24 is mainly CuAg. Since bonding interfaces are first formed at the periphery of the bonding region, the IMCs naturally grow at the periphery first. Region 4 is closest to the periphery of the bonding interface, so its IMC is formed earlier than the first three regions, and after a longer period of time, the Cu atoms in region 4 combine with the previously formed compounds to form a more stable CuAg_2,_ namely CuAg. Kirkendall voids can be seen in the upper-left corner of Figure 6d, but their size and scale are much smaller than that of Pd100-bonded sample.

In summary, the Ag layers on the pad surface of the Pd120-bonded sample are all involved in the formation of IMCs after 96 h of storage at 150 °C in a high-temperature environment. The IMCs are distributed in stripes without faults at the bonding interface, and their main components are CuAg_4_, CuAg_2_, CuAg, CuAg_3_, etc. In addition, no Pd atoms are found in any of the regions where the overall IMCs are located. Based on the location of the selected four regions and their respective IMC compositions, it is speculated that the formation order of IMCs is to first form CuAg_4_, then CuAg_2_, and then finally form stable CuAg. This is based on the fact that the Cu atoms diffuse rapidly into the Ag layer when the bonding interface begins to form, and because of the high content of Ag atoms, Ag-rich compounds (CuAg_4_, etc.) are formed first. Then, as the Cu atoms continue to diffuse, the compound undergoes a phase transition, gradually forming CuAg_2_, CuAg, and so on. The IMCs of Pd100 and Pd120 are organized in Table 3.

**Figure 7:**
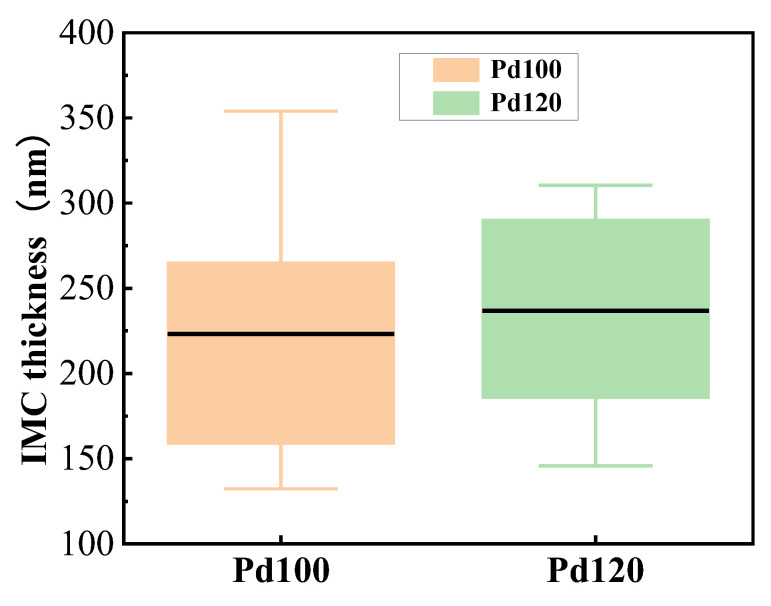
IMC thickness of bonded sample of Pd100 and Pd120.

Figure 7 shows that the maximum IMC thickness of the Pd100-bonded sample is significantly greater than that of the Pd120-bonded sample, while the average thickness is smaller, and the data for the Pd100-bonded sample has higher discreteness. Because the Pd100-bonded sample lacks the inhibitory effect of the Pd element on the diffusion of Cu atoms, Cu diffuses rapidly in some regions and the Kirkendall effect is significant. In the early stage of IMC growth, many pores and cracks are generated, which isolates the contact between Cu and Ag, thereby hindering the further expansion of IMCs. Therefore, the thickness of IMCs in some areas of the Pd100-bonded sample is very thin. The IMCs in a well-contacted region at the bonding interface can grow sufficiently to form a greater thickness. The Pd120-bonded sample shows slower growth and more uniform IMC size because of the high Pd content, and has not yet reached the maximum thickness after 96 h of holding time.

## 4. Conclusions

(1) Compared to Pd120-bonded sample, the Kirkendall effect is more pronounced at the bonding interface of the Pd100-bonded sample, with larger pore and crack sizes. The thickness of the IMC is uneven, and it is mainly composed of CuAg_2_ and CuAg. In contrast, the bonding interface of the Pd120-bonded sample has almost no microcracks, and the Kirkendall voids are small. The IMCs are uniform and relatively thin, with the main components being CuAg_4_, CuAg_2_, and CuAg.
(2) Pd was not detected in all of the IMCs, indicating that Pd is not involved in their formation and growth. The formation sequence of the IMCs begins with Cu atoms diffusing into the Ag layer to form a silver-rich compound, CuAg_4_, and the diffusion of Cu elements increases to form CuAg_2_, which finally forms CuAg. The Pd element can significantly weaken the Kirkendall effect and slow down the growth of Cu-Ag IMCs.
(3) IMCs grow rapidly in areas with low Pd content, resulting in pores and microcracks that may cause delamination at the bonding interface, whichwill hinder the diffusion of Cu and Ag atoms, and inhibit the sustained growth of IMCs.

## Figures and Tables

**Table 1:** The elemental composition of points in different regions and atomic content.

Detection Position	Cu (at.%)	Ag (at.%)	Pd (at.%)	Detection Position	Cu (at.%)	Ag (at.%)	Pd (at.%)
1	32.27	67.14	0	18	32.18	67.63	0
2	32.31	67.14	0	19	50.81	48.57	0
3	32.65	65.37	0	20	47.5	52.05	0
4	32.35	67.14	0	21	47.3	52.07	0
5	53.82	45.21	0	22	42.93	56.99	0
6	98.88	0.67	0	23	44.72	55.12	0
7	93.28	5.34	0.14	24	44.99	54.52	0
8	99.72	0.1	0.1	25	47.75	51.88	0
9	98.37	0.51	1.04	26	42.49	57.22	0
10	99.5	0.3	0.03	27	91.29	1.66	0.03
11	98.3	0.47	1.17	28	99.45	0.29	0.06
12	98.04	0.93	0.96	29	94.73	2.24	0
13	98.05	0.47	1.27	30	91.9	3.47	0
14	98.07	0.52	1.24	31	49.46	49.95	0
15	31.56	67.97	0	32	48.53	50.86	0
16	31.73	68.56	0	33	49.14	50.01	0
17	31.38	68.31	0	34	49.34	50.04	0

**Table 2:** The elemental composition and atomic content of each point.

Detection Position	Cu (at.%)	Ag (at.%)	Pd (at.%)	Detection Position	Cu (at.%)	Ag (at.%)	Pd (at.%)
1	98.07	0.71	1.22	13	27.33	72.67	0
2	43.26	56.74	0	14	24.46	75.54	0
3	19.39	80.61	0	15	99.22	0.77	0.01
4	20.28	79.72	0	16	99.34	0.65	0.01
5	19.74	80.26	0	17	79.31	20.69	0
6	19.01	80.99	0	18	18.72	81.28	0
7	19.26	80.74	0	19	18.85	81.15	0
8	19.68	80.32	0	20	18.91	81.09	0
9	100	0	0	21	37.16	62.84	0
10	99.72	0.28	0	22	32.08	66.97	0
11	99.88	0.12	0	23	45.93	54.05	0
12	25.37	74.63	0	24	46.3	53.7	0

**Table 3:** Comparison of the bonded sample of the Pd100 and Pd120 IMCs.

	Micropores	IMC Fault	IMC Cracks	Main Components of IMC
Pd100	More	existence	existence	CuAg_2_, CuAg
Pd120	Less	Less	Less	CuAg_4_, CuAg_2_, CuAg

## Data Availability

The original contributions presented in the study are included in the article, further inquiries can be directed to the corresponding author.
